# Crystal structure, synthesis and thermal properties of bis­(4-benzoyl­pyridine-κ*N*)bis­(iso­thio­cyanato-κ*N*)bis­(methanol-κ*N*)iron(II)

**DOI:** 10.1107/S2056989020001152

**Published:** 2020-01-31

**Authors:** Carsten Wellm, Christian Näther

**Affiliations:** a Institut für Anorganische Chemie, Universität Kiel, Max-Eyth. Str. 2, 24118 Kiel, Germany

**Keywords:** crystal structure, iron(II) thio­cyanate, solvate, discrete complex, hydrogen bonding

## Abstract

In the crystal structure of the title compound, [Fe(NCS)_2_(C_12_H_9_NO)_2_(CH_4_O)_2_], the Fe^II^ cations are octa­hedrally coordinated by two N atoms of 4-benzoyl­pyridine ligands, two N atoms of two terminal iso­thio­cyanate anions and two methanol mol­ecules into discrete complexes that are located on centres of inversion. These complexes are linked *via* inter­molecular O—H⋯O hydrogen bonds between the methanol O—H H atoms and the carbonyl O atoms of the 4-benzoyl­pyridine ligands, forming layers parallel to (101). Powder X-ray diffraction proved that a pure sample was obtained but that this compound is unstable and transforms into an unknown crystalline phase within several weeks. However, the solvent mol­ecules can be removed by heating in a thermobalance, which for the aged sample as well as the title compound leads to the formation of a compound with the composition Fe(NCS)_2_(4-benzoyl­pyridine)_2_, which exhibits a powder pattern that is similar to that of Mn(NCS)_2_(4-benzoyl­pyridine)_2_.

## Abstract 2

In the crystal structure of the title compound, the Fe^II^ cations are octa­hedrally coordinated into discrete complexes that are linked into layers *via* inter­molecular O—H⋯O hydrogen bonding.

## Chemical context   

The synthesis of new coordination compounds is still an important topic in modern coordination chemistry. In most cases, such compounds are prepared in solution but there are alternative routes such as, for example, mol­ecular milling or synthesis in molten flux (Braga *et al.*, 2005 ▸, 2006 ▸; James *et al.*, 2012 ▸; Höller *et al.*, 2010 ▸; Schönfeld *et al.*, 2012 ▸). In this context, thermal annealing is also of inter­est, especially for precursors that contain volatile ligands. This approach has been proven to be particularly useful for the synthesis of thio­cyanate coord­ination polymers, in which the metal cations are linked by the anionic ligands, because such compounds are frequently difficult to prepare in solution because their counterparts with terminally N-bonded anionic ligands are usually more stable (Näther *et al.*, 2013 ▸). This is one of the reasons why we became inter­ested in this class of compounds several years ago. In most cases, our precursors consist of discrete complexes, in which the metal cations are octa­hedrally coordinated by two terminal N-bonded thio­cyanate anions and four pyridine-based co-ligands. If the described compounds are heated, the co-ligands are removed in a stepwise manner, which enforces the formation of compounds with bridging anions because the octa­hedral coordination is usually retained (Näther *et al.*, 2013 ▸). One additional advantage of this approach is the fact that frequently different polymorphs or isomers can be obtained, if compared to the synthesis from solution (Wöhlert *et al.*, 2014 ▸; Werner *et al.*, 2015 ▸; Neumann *et al.*, 2018*a* ▸), which might be traced back to the fact that this anionic ligand exhibits a large structural variety (Mautner *et al.*, 2016*a* ▸,*b* ▸, 2018 ▸). Following this approach, in most cases compounds are obtained in which the metal cations are octa­hedrally coordinated by two N- and two S-bonding thio­cyanate anions as well as two coligands, all of them in *trans*-positions, and are linked into chains by pairs of μ-1,3-bridging anions (Neumann *et al.*, 2019 ▸; Rams *et al.*, 2020 ▸; Mautner *et al.*, 2018 ▸). However, in some cases a *cis*–*cis*–*trans* coordination is observed, which can lead to the formation of linear but also to corrugated chains (Jochim *et al.*, 2018 ▸; Neumann *et al.*, 2020 ▸).
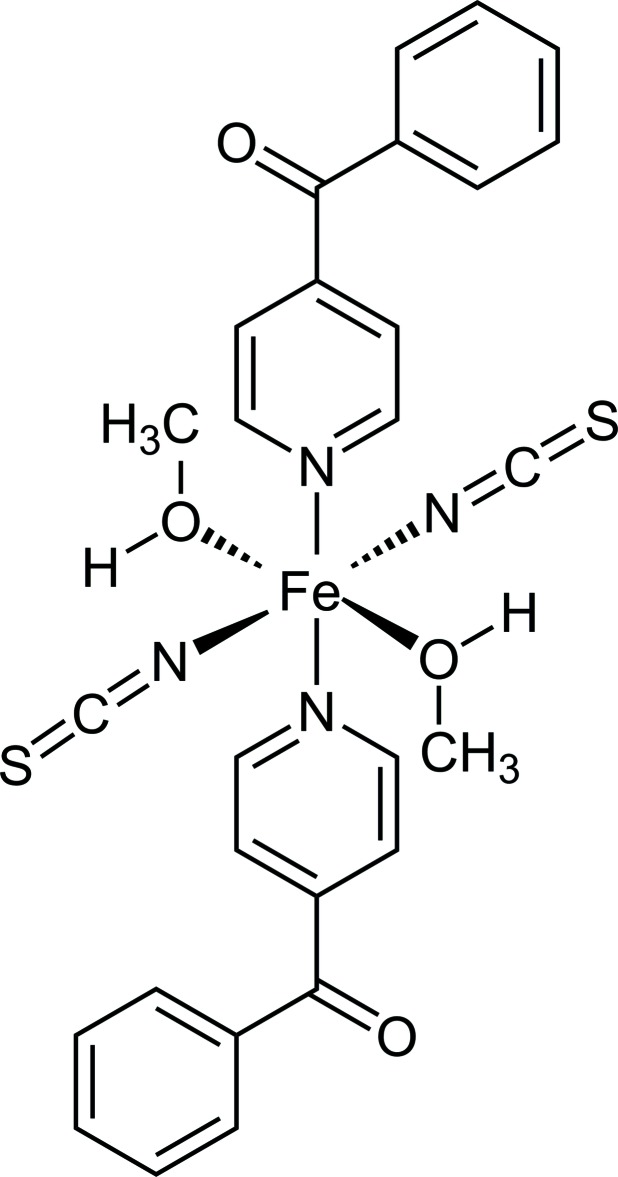


**Figure 1:**
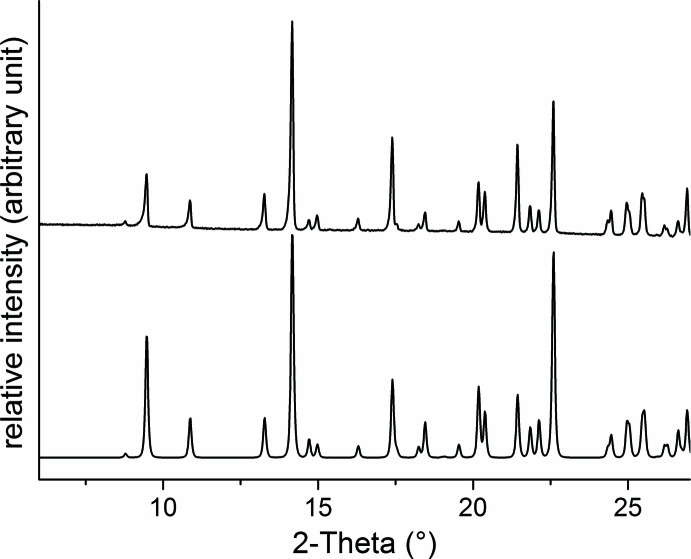
Experimental (top) and calculated (bottom) powder pattern of the title compound.

This is the case for *e.g.* [*M*(NCS)_2_(4-benzoyl­pyridine)_2_]_*n*_ (*M* = Co, Ni), in which the two N and two S atoms of the bridging anionic ligands are in *cis*-positions, whereas the two apical 4-benzoyl­pyridine ligands are *trans*-coordinating (Rams *et al.*, 2017 ▸; Jochim *et al.*, 2018 ▸). This is in contrast to the corresponding Cd compound, in which the Cd^II^ cations shows the usual all-*trans* coordination (Neumann *et al.*, 2018*b* ▸). In this context, we became inter­ested in the corresponding compounds based on Mn^II^ and Fe^II^. However, the compounds with bridging anionic ligands are not available from solution and therefore, we tried to prepare them by thermal decomposition of discrete complexes with the composition *M*(NCS)_2_(4-benzoyl­pyridine)_4_ (*M* = Mn and Fe; Wellm *et al.*, 2018 ▸, 2019*a* ▸). Unfortunately, the X-ray powder pattern of the residues are of low quality and don’t seem to be isotypic to the Co^II^, Ni^II^ or Cd^II^ phases. Therefore, we looked for a precursor that consists of two different coligands, one of which is more volatile and we found that, with methanol as solvent, crystals with the composition Fe(NCS)_2_(4-benzoyl­pyridine)_2_(CH_3_OH)_2_ can be obtained. Comparison of the experimental XRPD patterns with that calculated from single-crystal data proves that a pure phase has been obtained (Fig. 1 ▸). We also have found that on storage over weeks, the title compound transforms into a new crystalline phase that exhibits a powder pattern completely different from that of the title compound (see Figure S1 in the supporting information). Compared to the title compound, the CN stretching vibration of the thio­cyanate anions is shifted from 2050 cm^−1^ to 2074 cm^−1^, and from thermogravimetric measurements it is indicated that about half of the methanol mol­ecules are removed (Figure S2). If the solvent is removed completely from this crystalline phase, the CN stretching vibration shifts to 2084 cm^−1^ and a powder pattern is observed that cannot be indexed and that is different from those calculated for the known phases of [*M*(NCS)_2_(4-benzoyl­pyridine)_2_]_*n*_ (*M* = Co, Ni, Cd; Figure S1). However, if the title compound is heated in a thermobalance, two mass losses are observed that are in reasonable agreement with that calculated for the removal of the methanol mol­ecules in the first and the remaining 4-benzoyl­pyridine ligands in the second step (calculated: 10.6% and 60.5%). If the residue formed after methanol removal is investigated by XRPD, it is obvious that the same crystalline phase has been obtained that will form if the discrete complex Fe(NCS)_2_(4-benzoyl­pyridine)_4_ is thermally decomposed (Figures S1 and S2; Wellm, & Näther, 2019*a* ▸). There are some similarities to the pattern of the residue obtained by thermal decomposition of Mn(NCS)_2_(4-benzoyl­pyridine)_4_ (Wellm & Näther, 2018 ▸), but it is different from those calculated for [*M*(NCS)_2_(4-benzoyl­pyridine)_2_]_*n*_ (*M* = Co, Ni, Cd; Figure S1).

## Structural commentary   

**Figure 2:**
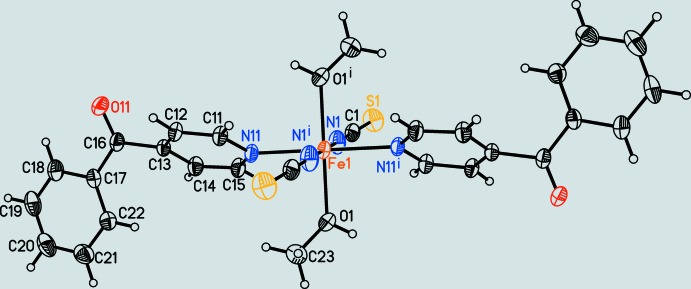
View of a discrete complex with atom labelling and displacement ellipsoids drawn at the 50% probability level. Symmetry code: (i) −*x*, −*y*, −*z* + 1.

The asymmetric unit of the title compound [Fe(NCS)_2_(C_12_H_9_NO)_2_(MeOH)_2_] consists of one terminal N-bonded thio­cyanate anion, one O-bonded methanol and one N-bonded 4-benzoyl­pyridine ligand in general positions and one Fe^II^ cation located on a centre of inversion (Fig. 2 ▸). The Fe^II^ cation is octa­hedrally coordinated by two thio­cyanate anions, two methanol and two 4-benzoyl­pyridine ligands, with each pair of the same ligand in the *trans*-position. The Fe—N bond length to the 4-benzoyl­pyridine ligand [2.2270 (12) Å] is longer than that to the thio­cyanate anion [2.0823 (15) Å] (Table 1 ▸). From the bond angles around the metal centers as well as the value for the angle variance (0.93) and the quadratic elongation (1.002) calculated by a procedure published by Robinson *et al.* (1971 ▸), it is obvious that the octa­hedra are slightly distorted. The 4-benzoyl­pyridine ligands are not coplanar as demonstrated by the values of the dihedral angles between the pyridine ring (N11/C11–C15) and the carbonyl group (C13/C16/C17/O11) of 47.9 (1)° and between the carbonyl group (C13/C16/ C17/O11) and the phenyl ring (C17–C22) of 16.6 (1)°.

## Supra­molecular features   

**Figure 3:**
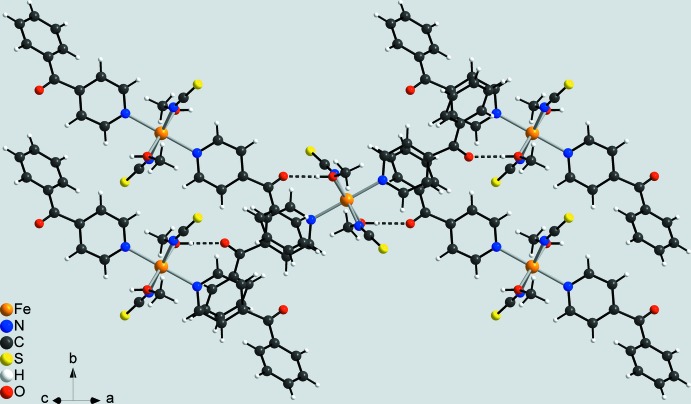
Crystal packing of the title compound viewed along (101) with inter­molecular O—H⋯O hydrogen bonding shown as dashed lines.

**Figure 4:**
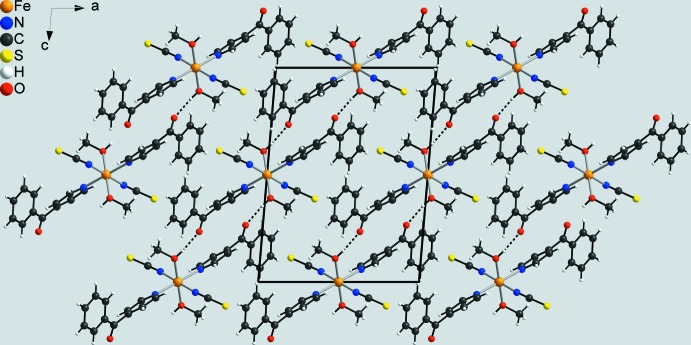
Crystal packing of the title compound viewed along the crystallographic *b* axis with inter­molecular O—H⋯O hydrogen bonding shown as dashed lines.

In the crystal of the title compound, the discrete complex mol­ecules are linked by inter­molecular O—H⋯O hydrogen bonds between the hydroxyl H atom of the methanol ligand and the carbonyl oxygen atom of a 4-benzoyl­pyridine ligand of a neighbouring complex (Table 2 ▸). Each of the complexes are linked to four symmetry-equivalent complexes into layers parallel to (101) (Fig. 3 ▸). Between these layers, no pronounced inter­molecular inter­actions are observed (Fig. 4 ▸).

## Database survey   

According to the Cambridge Structural Database (CSD, version 5.40, updated Feb. 2019; Groom *et al.*, 2016 ▸), several compounds based on 4-benzoyl­pyridine and transition-metal thio­cyanates have been reported. This includes one square-planar copper complex with the composition [Cu(NCS)_2_(4-benzoyl­pyridine)_2_] (Bai *et al.*, 2011 ▸) and the Zn complex [Zn(NCS)_2_(4-benzoyl­pyridine)_2_], in which the Zn^II^ cations are tetra­hedrally coordinated (Neumann *et al.*, 2018*b* ▸). In all of the remaining compounds the metal cations are octa­hedrally coordinated. Some of them are coordination polymers with the general composition [*M*(NCS)_2_(4-benzoyl­pyridine)_2_]_*n*_ (*M* = Cd^II^, Ni^II^, Co^II^), in which the metal centres are bridged by pairs of μ-1,3-coordinating thio­cyanate anions into chains (Neumann *et al.*, 2018*b* ▸; Rams *et al.*, 2017 ▸; Jochim *et al.*, 2018 ▸). The remaining compounds are octa­hedrally coordinated complexes with two terminal thio­cyanate anions and either four 4-benzoyl­pyridine ligands or two 4-benzoyl­pyridine ligands and two additional solvate ligands (Drew *et al.*, 1985 ▸; Neumann *et al.*, 2018*b* ▸; Soliman *et al.*, 2014 ▸; Suckert *et al.*, 2017*a* ▸,*b* ▸; Wellm & Näther, 2018 ▸, 2019*a* ▸,*b* ▸,*c* ▸).

## Synthesis and crystallization   

FeCl_2_·4 H_2_O and KSCN were purchased from Merck and 4-benzoyl­pyridine was purchased from Alfa Aesar.

**Synthesis:**

Crystals of the title compound suitable for single-crystal X-ray diffraction were obtained by the reaction of 59.6 mg FeCl_2_·4H_2_O (0.3 mmol) and 58.3 mg of KSCN (0.6 mmol) with 27.5 mg of 4-benzoyl­pyridine (0.15 mmol) in methanol (1.5 mL) within a few days.

For the synthesis of larger amounts of a polycrystalline powder, 398 mg of FeCl_2_·4H_2_O (2 mmol) and 396 mg of KSCN (4 mmol) were stirred in methanol (2 mL) for 16 h and the precipitating KCl was filtered off and washed two times with methanol (0.5 mL). 366 mg of (2 mmol) 4-benzoyl­pyridine were added and this reaction mixture was stirred for four days. The product was filtered off and directly analysed, because it proved to be unstable at room temperature if stored for a longer time.

**Experimental details:**

Differential thermoanalysis and thermogravimetry (DTA–TG) was performed in a dynamic nitro­gen atmosphere in Al_2_O_3_ crucibles using an STA PT1600 thermobalance from Linseis. The XRPD measurements were performed using a Stoe Transmission Powder Diffraction System (STADI P) with Cu *K*α radiation that was equipped with a linear position-sensitive MYTHEN detector from STOE & Cie. The IR data were measured using a Bruker Alpha-P ATR-IR Spectrometer.

## Refinement   

Crystal data, data collection and structure refinement details are summarized in Table 3 ▸. The C—H H atoms were positioned with idealized geometry and were refined with fixed isotropic displacement parameters *U*_iso_(H) = 1.2 *U*_eq_(C) for aromatic and *U*_iso_(H) = 1.5 *U*_eq_(C) for methyl H atoms using a riding model. The O—H H atom was located in a difference map, its bond length was set to an ideal value of 0.84 Å and finally, it was refined with *U*_iso_(H) = 1.5 *U*_eq_(O) using a riding model.

## Supplementary Material

Crystal structure: contains datablock(s) I. DOI: 10.1107/S2056989020001152/lh5944sup1.cif

Structure factors: contains datablock(s) I. DOI: 10.1107/S2056989020001152/lh5944Isup2.hkl

FigureS1. Experimental powder patterns of the title compound (A), of the title compound kept at room temperature for two months (B), of the residue obtained after the first mass loss in a TG measurements of the aforementioned aged sample (C), of the residue obtained after the first mass loss in a TG measurements of the title compound (D), of the residue obtained after the first mass loss in a TG measurement of [Fe(NCS)2(4-benzoylpyridine)4] (E),of the residue obtained after the first mass loss in a TG measurement of [Mn(NCS)2(4-benzoylpyridine)4] (F) and the calculated patterns of [Co(NCS)2(4-benzoylpyridine)2] (G) and [Cd(NCS)2(4-benzoylpyridine)2] (H). DOI: 10.1107/S2056989020001152/lh5944sup3.tif

FigureS2. IR spectra of the of the title compound (A), of the title compound kept at room temperature for two months (B), of the residue obtained after the first mass loss in a TG measurements of the aforementioned aged sample (C), of the residue obtained after the first mass loss in a TG measurements of the title compound (D), of the residue obtained after the first mass loss in a TG measurement of [Fe(NCS)2(4-benzoylpyridine)4] (E), of the residue obtained after the first mass loss in a TG measurement of [Mn(NCS)2(4-benzoylpyridine)4] (F), of [Co(NCS)2(4-benzoylpyridine)2] (G) and of [Cd(NCS)2(4-benzoylpyridine)2] (H). DOI: 10.1107/S2056989020001152/lh5944sup4.tif

FigureS3. DTG, TG and DTA curves of the title compound at a heating rate of 1 C/min with the experimental mass loss in % and peak temperatures in C. DOI: 10.1107/S2056989020001152/lh5944sup5.tif

CCDC reference: 1980369

Additional supporting information:  crystallographic information; 3D view; checkCIF report

## Figures and Tables

**Table 1:** Selected geometric parameters (Å, °)

Fe1—N1^i^	2.0823 (15)	Fe1—O1	2.1780 (12)
Fe1—N1	2.0823 (15)	Fe1—N11^i^	2.2270 (12)
Fe1—O1^i^	2.1780 (12)	Fe1—N11	2.2270 (12)
			
N1^i^—Fe1—N1	180.00 (8)	O1^i^—Fe1—N11^i^	88.57 (5)
N1^i^—Fe1—O1^i^	89.31 (6)	O1—Fe1—N11^i^	91.43 (5)
N1—Fe1—O1^i^	90.69 (6)	N1^i^—Fe1—N11	90.13 (5)
N1^i^—Fe1—O1	90.69 (6)	N1—Fe1—N11	89.87 (5)
N1—Fe1—O1	89.31 (6)	O1^i^—Fe1—N11	91.43 (5)
O1^i^—Fe1—O1	180.00 (4)	O1—Fe1—N11	88.57 (5)
N1^i^—Fe1—N11^i^	89.87 (5)	N11^i^—Fe1—N11	180.0
N1—Fe1—N11^i^	90.13 (5)		

Symmetry code: (i) 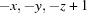.

**Table 2:** Hydrogen-bond geometry (Å, °)

*D*—H⋯*A*	*D*—H	H⋯*A*	*D*⋯*A*	*D*—H⋯*A*
O1—H1⋯O11^ii^	0.84	1.92	2.7574 (16)	174

Symmetry code: (ii) 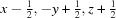.

**Table 3:** Experimental details

Crystal data	
Chemical formula	[Fe(NCS)_2_(C_12_H_9_NO)_2_(CH_4_O)_2_]
*M*_r_	602.50
Crystal system, space group	Monoclinic, *P*2_1_/*n*
Temperature (K)	200
*a*, *b*, *c* (Å)	12.1111 (8), 7.2385 (3), 16.1716 (12)
β (°)	94.730 (8)
*V* (Å^3^)	1412.87 (15)
*Z*	2
Radiation type	Mo *K*α
μ (mm^−1^)	0.72
Crystal size (mm)	0.12 × 0.08 × 0.06

Data collection	
Diffractometer	STOE *IPDS1*
Absorption correction	Numerical (*X-SHAPE* and *X-RED32*; Stoe & Cie, 2008 ▸)
*T*_min_, *T*_max_	0.834, 0.973
No. of measured, independent and observed [*I* > 2σ(*I*)] reflections	19758, 3422, 3012
*R*_int_	0.038
(sin θ/λ)_max_ (Å^−1^)	0.663

Refinement	
*R*[*F* ^2^ > 2σ(*F* ^2^)], *wR*(*F* ^2^), *S*	0.037, 0.104, 1.03
No. of reflections	3422
No. of parameters	179
H-atom treatment	H-atom parameters constrained
Δρ_max_, Δρ_min_ (e Å^−3^)	0.36, −0.51

Computer programs: *X-AREA* (Stoe & Cie, 2008 ▸), *SHELXS97* (Sheldrick, 2008 ▸), *SHELXL2014* (Sheldrick, 2015 ▸), *XP* in *SHELXTL* (Sheldrick, 2008 ▸) and *DIAMOND* (Brandenburg, 1999 ▸) and *publCIF* (Westrip, 2010 ▸).

## supplementary crystallographic information

### Crystal data

**Table:**

[Fe(NCS)_2_(C_12_H_9_NO)_2_(CH_4_O)_2_]	*F*(000) = 624
*M**_r_* = 602.50	*D*_x_ = 1.416 Mg m^−^^3^
Monoclinic, *P*2_1_/*n*	Mo *K*α radiation, λ = 0.71073 Å
*a* = 12.1111 (8) Å	Cell parameters from 19758 reflections
*b* = 7.2385 (3) Å	θ = 2.5–28.1°
*c* = 16.1716 (12) Å	µ = 0.72 mm^−^^1^
β = 94.730 (8)°	*T* = 200 K
*V* = 1412.87 (15) Å^3^	Block, light yellow
*Z* = 2	0.12 × 0.08 × 0.06 mm

### Data collection

**Table:**

STOE IPDS-1 diffractometer	3012 reflections with *I* > 2σ(*I*)
Phi scans	*R*_int_ = 0.038
Absorption correction: numerical (X-SHAPE and X-RED32; Stoe & Cie, 2008)	θ_max_ = 28.1°, θ_min_ = 2.5°
*T*_min_ = 0.834, *T*_max_ = 0.973	*h* = −16→16
19758 measured reflections	*k* = −9→9
3422 independent reflections	*l* = −21→21

### Refinement

**Table:**

Refinement on *F*^2^	Hydrogen site location: mixed
Least-squares matrix: full	H-atom parameters constrained
*R*[*F*^2^ > 2σ(*F*^2^)] = 0.037	*w* = 1/[σ^2^(*F*_o_^2^) + (0.0694*P*)^2^ + 0.430*P*] where *P* = (*F*_o_^2^ + 2*F*_c_^2^)/3
*wR*(*F*^2^) = 0.104	(Δ/σ)_max_ < 0.001
*S* = 1.03	Δρ_max_ = 0.36 e Å^−^^3^
3422 reflections	Δρ_min_ = −0.51 e Å^−^^3^
179 parameters	Extinction correction: SHELXL, Fc^*^=kFc[1+0.001xFc^2^λ^3^/sin(2θ)]^-1/4^
0 restraints	Extinction coefficient: 0.033 (3)

### Special details

**Table:**

Geometry. All esds (except the esd in the dihedral angle between two l.s. planes) are estimated using the full covariance matrix. The cell esds are taken into account individually in the estimation of esds in distances, angles and torsion angles; correlations between esds in cell parameters are only used when they are defined by crystal symmetry. An approximate (isotropic) treatment of cell esds is used for estimating esds involving l.s. planes.

### Fractional atomic coordinates and isotropic or equivalent isotropic displacement parameters (Å^2^)

**Table:**

	*x*	*y*	*z*	*U*_iso_*/*U*_eq_	
Fe1	0.0000	0.0000	0.5000	0.02289 (12)	
N1	−0.11410 (12)	0.1948 (2)	0.45147 (10)	0.0371 (3)	
C1	−0.19503 (13)	0.2661 (2)	0.42451 (10)	0.0281 (3)	
S1	−0.30854 (4)	0.36594 (7)	0.38796 (3)	0.04332 (15)	
N11	0.13430 (11)	0.14836 (18)	0.44116 (8)	0.0258 (3)	
C11	0.21746 (13)	0.0529 (2)	0.41200 (10)	0.0277 (3)	
H11	0.2228	−0.0756	0.4236	0.033*	
C12	0.29604 (13)	0.1328 (2)	0.36572 (9)	0.0270 (3)	
H12	0.3521	0.0594	0.3442	0.032*	
C13	0.29163 (12)	0.3217 (2)	0.35128 (9)	0.0248 (3)	
C14	0.20752 (13)	0.4230 (2)	0.38307 (10)	0.0298 (3)	
H14	0.2031	0.5529	0.3752	0.036*	
C15	0.13013 (13)	0.3312 (2)	0.42649 (10)	0.0301 (3)	
H15	0.0715	0.4006	0.4468	0.036*	
C16	0.37576 (13)	0.4045 (2)	0.29890 (9)	0.0270 (3)	
C17	0.43593 (12)	0.5737 (2)	0.32626 (10)	0.0264 (3)	
C18	0.49691 (14)	0.6691 (2)	0.27018 (11)	0.0346 (4)	
H18	0.4953	0.6297	0.2141	0.042*	
C19	0.55965 (16)	0.8209 (3)	0.29656 (14)	0.0433 (4)	
H19	0.6001	0.8871	0.2583	0.052*	
C20	0.56366 (16)	0.8766 (3)	0.37870 (15)	0.0450 (5)	
H20	0.6074	0.9802	0.3967	0.054*	
C21	0.50409 (16)	0.7819 (3)	0.43474 (13)	0.0399 (4)	
H21	0.5075	0.8202	0.4911	0.048*	
C22	0.43953 (13)	0.6315 (2)	0.40878 (10)	0.0310 (3)	
H22	0.3978	0.5679	0.4471	0.037*	
O11	0.39260 (11)	0.32281 (19)	0.23480 (7)	0.0377 (3)	
C23	0.1420 (2)	0.2043 (4)	0.64942 (14)	0.0593 (6)	
H23A	0.1367	0.2868	0.6970	0.089*	
H23B	0.1741	0.0861	0.6686	0.089*	
H23C	0.1892	0.2611	0.6101	0.089*	
O1	0.03337 (11)	0.1736 (2)	0.60921 (7)	0.0384 (3)	
H1	−0.0132	0.1745	0.6449	0.058*	

### Atomic displacement parameters (Å^2^)

**Table:**

	*U*^11^	*U*^22^	*U*^33^	*U*^12^	*U*^13^	*U*^23^
Fe1	0.01880 (17)	0.02661 (18)	0.02419 (17)	0.00042 (10)	0.00730 (11)	0.00354 (10)
N1	0.0278 (7)	0.0385 (8)	0.0454 (8)	0.0046 (6)	0.0063 (6)	0.0136 (6)
C1	0.0294 (7)	0.0260 (7)	0.0300 (7)	−0.0012 (6)	0.0080 (6)	0.0049 (6)
S1	0.0333 (2)	0.0431 (3)	0.0525 (3)	0.00961 (18)	−0.00278 (19)	0.0089 (2)
N11	0.0226 (6)	0.0289 (6)	0.0271 (6)	−0.0030 (5)	0.0089 (5)	0.0019 (5)
C11	0.0258 (7)	0.0269 (7)	0.0316 (7)	−0.0003 (6)	0.0096 (6)	0.0031 (6)
C12	0.0232 (7)	0.0290 (7)	0.0301 (7)	0.0008 (6)	0.0104 (6)	0.0018 (6)
C13	0.0222 (7)	0.0290 (7)	0.0238 (6)	−0.0045 (6)	0.0054 (5)	0.0017 (5)
C14	0.0286 (8)	0.0247 (7)	0.0375 (8)	−0.0009 (6)	0.0104 (6)	0.0014 (6)
C15	0.0259 (7)	0.0277 (7)	0.0382 (8)	−0.0002 (6)	0.0129 (6)	−0.0008 (6)
C16	0.0239 (7)	0.0313 (7)	0.0264 (7)	−0.0013 (6)	0.0063 (5)	0.0059 (6)
C17	0.0199 (6)	0.0281 (7)	0.0313 (7)	0.0001 (6)	0.0034 (5)	0.0058 (6)
C18	0.0310 (8)	0.0350 (8)	0.0388 (8)	−0.0036 (7)	0.0082 (7)	0.0086 (7)
C19	0.0333 (9)	0.0351 (9)	0.0625 (12)	−0.0087 (7)	0.0109 (8)	0.0111 (8)
C20	0.0318 (9)	0.0300 (8)	0.0730 (14)	−0.0049 (7)	0.0034 (9)	−0.0040 (8)
C21	0.0357 (9)	0.0342 (9)	0.0494 (10)	0.0004 (7)	0.0008 (8)	−0.0081 (8)
C22	0.0274 (7)	0.0316 (8)	0.0341 (8)	−0.0002 (6)	0.0035 (6)	0.0004 (6)
O11	0.0429 (7)	0.0434 (7)	0.0288 (6)	−0.0114 (6)	0.0157 (5)	−0.0027 (5)
C23	0.0489 (12)	0.0856 (17)	0.0439 (11)	−0.0272 (12)	0.0077 (9)	−0.0189 (11)
O1	0.0357 (6)	0.0517 (8)	0.0293 (6)	−0.0068 (6)	0.0123 (5)	−0.0080 (5)

### Geometric parameters (Å, º)

**Table:**

Fe1—N1^i^	2.0823 (15)	C16—O11	1.225 (2)
Fe1—N1	2.0823 (15)	C16—C17	1.474 (2)
Fe1—O1^i^	2.1780 (12)	C17—C22	1.396 (2)
Fe1—O1	2.1780 (12)	C17—C18	1.398 (2)
Fe1—N11^i^	2.2270 (12)	C18—C19	1.383 (3)
Fe1—N11	2.2270 (12)	C18—H18	0.9500
N1—C1	1.160 (2)	C19—C20	1.385 (3)
C1—S1	1.6209 (17)	C19—H19	0.9500
N11—C11	1.339 (2)	C20—C21	1.386 (3)
N11—C15	1.345 (2)	C20—H20	0.9500
C11—C12	1.385 (2)	C21—C22	1.385 (2)
C11—H11	0.9500	C21—H21	0.9500
C12—C13	1.388 (2)	C22—H22	0.9500
C12—H12	0.9500	C23—O1	1.436 (3)
C13—C14	1.388 (2)	C23—H23A	0.9800
C13—C16	1.502 (2)	C23—H23B	0.9800
C14—C15	1.386 (2)	C23—H23C	0.9800
C14—H14	0.9500	O1—H1	0.8401
C15—H15	0.9500		
			
N1^i^—Fe1—N1	180.00 (8)	N11—C15—H15	118.5
N1^i^—Fe1—O1^i^	89.31 (6)	C14—C15—H15	118.5
N1—Fe1—O1^i^	90.69 (6)	O11—C16—C17	122.83 (14)
N1^i^—Fe1—O1	90.69 (6)	O11—C16—C13	116.92 (14)
N1—Fe1—O1	89.31 (6)	C17—C16—C13	120.23 (13)
O1^i^—Fe1—O1	180.00 (4)	C22—C17—C18	119.69 (15)
N1^i^—Fe1—N11^i^	89.87 (5)	C22—C17—C16	120.78 (14)
N1—Fe1—N11^i^	90.13 (5)	C18—C17—C16	119.34 (15)
O1^i^—Fe1—N11^i^	88.57 (5)	C19—C18—C17	119.90 (17)
O1—Fe1—N11^i^	91.43 (5)	C19—C18—H18	120.1
N1^i^—Fe1—N11	90.13 (5)	C17—C18—H18	120.1
N1—Fe1—N11	89.87 (5)	C18—C19—C20	120.13 (17)
O1^i^—Fe1—N11	91.43 (5)	C18—C19—H19	119.9
O1—Fe1—N11	88.57 (5)	C20—C19—H19	119.9
N11^i^—Fe1—N11	180.0	C19—C20—C21	120.28 (17)
C1—N1—Fe1	163.04 (14)	C19—C20—H20	119.9
N1—C1—S1	179.32 (16)	C21—C20—H20	119.9
C11—N11—C15	117.66 (13)	C22—C21—C20	120.13 (18)
C11—N11—Fe1	119.92 (10)	C22—C21—H21	119.9
C15—N11—Fe1	122.13 (10)	C20—C21—H21	119.9
N11—C11—C12	123.04 (15)	C21—C22—C17	119.86 (16)
N11—C11—H11	118.5	C21—C22—H22	120.1
C12—C11—H11	118.5	C17—C22—H22	120.1
C11—C12—C13	118.94 (14)	O1—C23—H23A	109.5
C11—C12—H12	120.5	O1—C23—H23B	109.5
C13—C12—H12	120.5	H23A—C23—H23B	109.5
C12—C13—C14	118.52 (13)	O1—C23—H23C	109.5
C12—C13—C16	117.99 (14)	H23A—C23—H23C	109.5
C14—C13—C16	123.44 (14)	H23B—C23—H23C	109.5
C15—C14—C13	118.83 (15)	C23—O1—Fe1	123.95 (12)
C15—C14—H14	120.6	C23—O1—H1	109.2
C13—C14—H14	120.6	Fe1—O1—H1	118.2
N11—C15—C14	122.95 (14)		

Symmetry code: (i) −*x*, −*y*, −*z*+1.

### Hydrogen-bond geometry (Å, º)

**Table:**

*D*—H···*A*	*D*—H	H···*A*	*D*···*A*	*D*—H···*A*
O1—H1···O11^ii^	0.84	1.92	2.7574 (16)	174

Symmetry code: (ii) *x*−1/2, −*y*+1/2, *z*+1/2.
