# Supplementary figures and images for: Crystal structure, synthesis and thermal properties of bis­(4-benzoyl­pyridine-κN)bis­(iso­thio­cyanato-κN)bis­(methanol-κN)iron(II)
Source: Acta Crystallogr E Crystallogr Commun. 2020 Jan 31;76(Pt 2):276–80. doi: 10.1107/S2056989020001152 (PMC7001824; doi:10.1107/S2056989020001152)

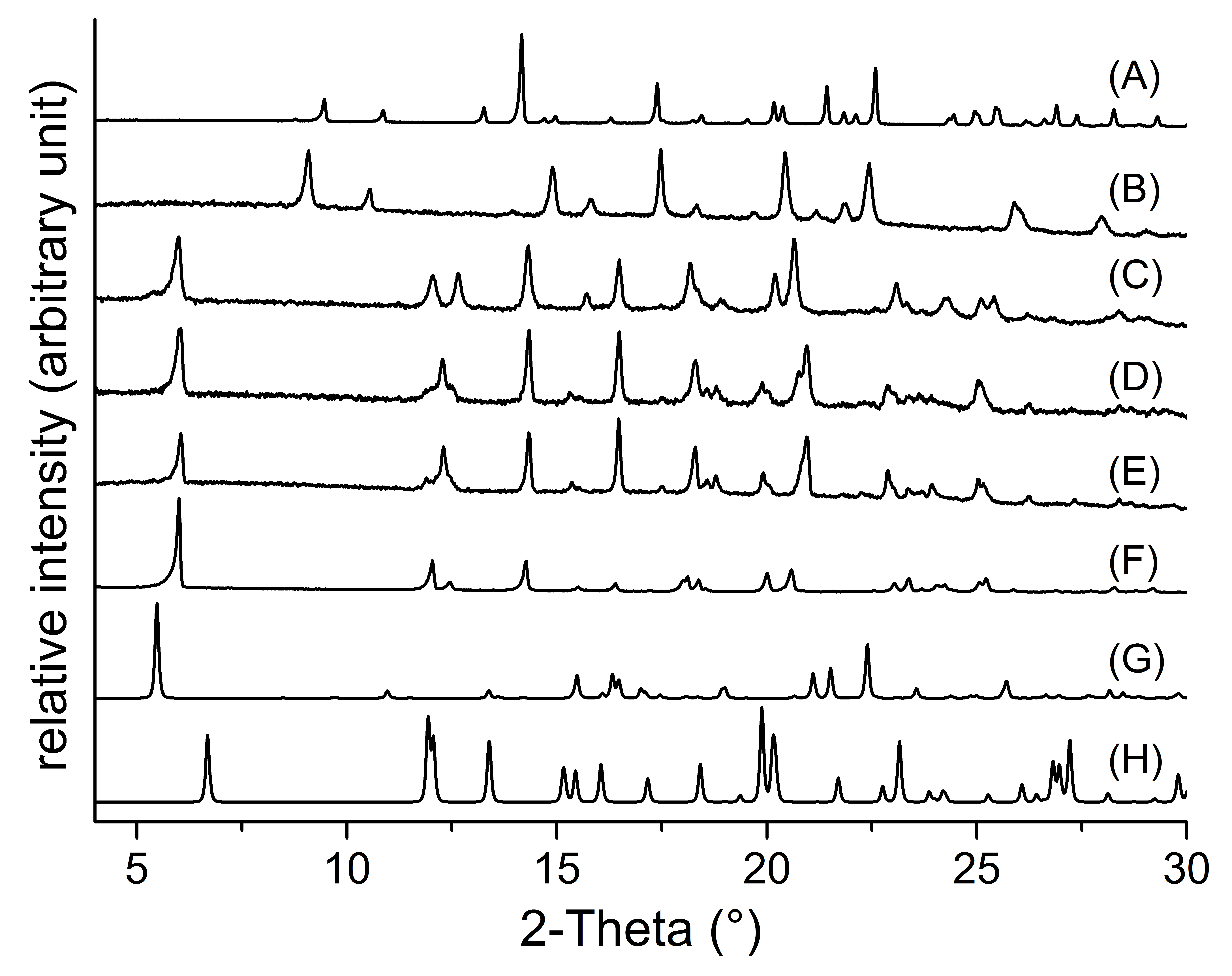

Supplement: Supplementary file 3 [file e-76-00276-sup3.tif]

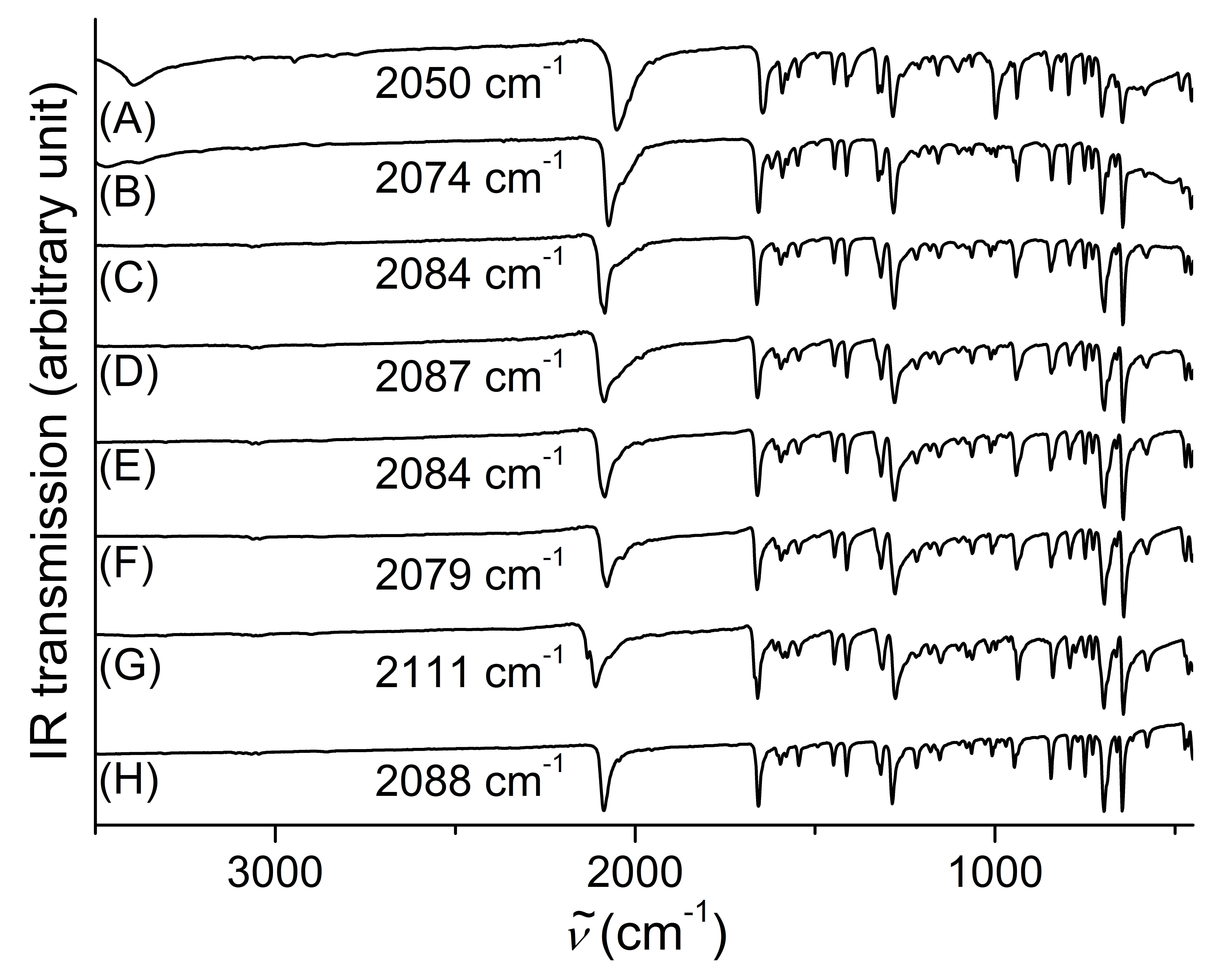

Supplement: Supplementary file 4 [file e-76-00276-sup4.tif]
